# Acute Myopericarditis After First Dose of mRNA-1273 SARS-CoV-2 Vaccine in a Young Adult

**DOI:** 10.7759/cureus.22111

**Published:** 2022-02-11

**Authors:** Kahtan Fadah, Mehran Abolbashari, Chandra Ojha, Haider Alkhateeb

**Affiliations:** 1 Internal Medicine, Texas Tech University Health Sciences Center, El Paso Paul L. Foster School of Medicine, El Paso, USA; 2 Internal Medicine, Texas Tech University Health Sciences Center, Division of Cardiovascular Medicine, El Paso, USA; 3 Cardiovascular Medicine, Texas Tech University Health Sciences Center, El Paso, USA

**Keywords:** side effects of medical treatment, covid 19, myo-pericarditis, young population, covid-19 vaccine

## Abstract

The coronavirus disease (COVID-19 or SARS-CoV-2) pandemic has brought the global community to a halt. A return to normalcy is dependent on effective reopening strategies that encourage herd immunity through the implementation of vaccines. Cardiopulmonary inflammation has been reported in SARS-CoV-2 infection, independent of the severity, mainly amongst the juvenile population. Cardiovascular involvement following SARS-CoV-2 infection is associated with higher mortality and morbidity. Cardiovascular complications following COVID-19 vaccination have been documented as less severe, with no link between cardiovascular injury and death. This case report describes the presentation of an otherwise healthy 18-year-old male who experienced retrosternal chest pain after receiving a first dose of the mRNA-1273 vaccine. The patient had a negative polymerase chain reaction (PCR) test for COVID-19 infection. An electrocardiogram revealed diffuse ST elevation and PR segment depression, with increased inflammatory markers consistent with pericarditis. Elevation of troponin (16 ng/mL), evidence of borderline reduced ejection fraction (50-55%), and global left ventricular hypokinesis were suggestive of myopericarditis. Infectious and autoimmune studies were negative. The patient was treated mainly with non-steroidal anti-inflammatory drugs and colchicine, which resulted in a significant improvement of clinical symptoms. As the administration of emergency COVID-19 vaccines continues worldwide, it is of paramount importance to be aware of possible adverse events, including those affecting the cardiovascular system.

## Introduction

Severe acute respiratory syndrome coronavirus 2 (SARS-CoV-2) was first recognized in December 2019 as a primary cause of severe respiratory tract infection. Cardiac involvement secondary to coronavirus disease is well established in the literature. It had been recognized by the American College of Cardiology as part of multi-organ involvement associated with coronavirus infection [1,2]. Cardiovascular inflammation has also been reported, independent of the severity of infection, with no major lung involvement, which suggests that the underlying mechanism is a direct immune injury in response to viral replication and dissemination within cardiac tissue [3]. The mRNA COVID-19 vaccines with two-dose regimens that are in use globally have been shown to confer 94-95% protection against severe coronavirus infection, with a promising safety profile [4]. Ammirati et al. reported a case of myocarditis that occurred three days after receiving a second dose of the BNT162b2 mRNA vaccine in a patient who had previously been infected with SARS-CoV-2 [2]. Here, we describe the case of an otherwise healthy 18-year-old man who presented with myopericarditis involvement three days after a first dose of the mRNA-1273 vaccine. There was no clinical or laboratory evidence of COVID-19 infection.

## Case presentation

**Figure 1:**
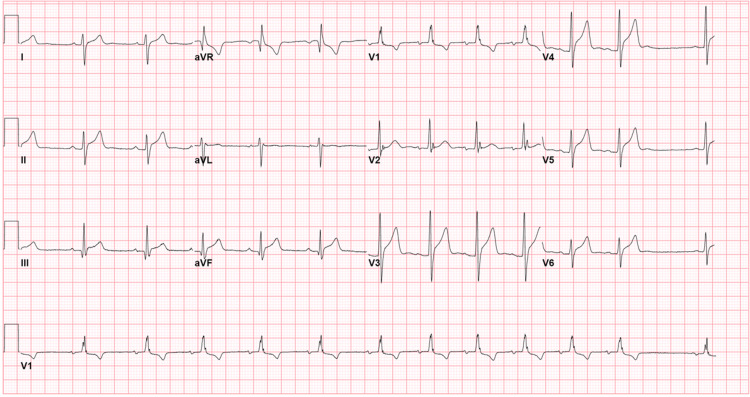
Diffuse ST elevation with diffuse PR segment depression and incomplete right bundle branch block

An 18-year-old male with no significant medical history presented with non-radiating chest pain that had progressively worsened in the 12 hours prior to admission. Prior to arrival, pain intensity had increased from 2/10 to 8/10. Other associated symptoms included bilateral finger numbness and significant lateral and occipital headache (which suddenly resolved prior to admission). The patient did not report any other symptoms or recent infection. The patient received his first COVID-19 vaccine three days prior to experiencing symptoms. He denied any smoking, recreational drug, or alcohol use. He was not taking any medication at home. At admission, the patient was afebrile at 37.1ºC, vitally stable (with oxygen saturation of 96% on room air), and with a heart rate 65 beats per minute, respiratory rate of 18 breaths per minute, blood pressure of 123/66 mmHg, and weight of 93.6 kg. Physical examination showed a regular heart rate and rhythm, with no murmur, gallop, peripheral edema, or jugular vein distention. He was found to have mild leukocytosis (13.67 × 103/mL), predominately neutrophilic, with no bandemia or eosinophilia. His leukocytosis resolved the next day. The troponin I level at admission was 6.1 ng/mL (normal range 0.000-0.034 ng/ml), trending upwards. The echocardiogram (EKG) showed diffuse ST elevation with diffuse PR segment depression, suggestive of pericarditis (Figure 1).

**Figure 2:**
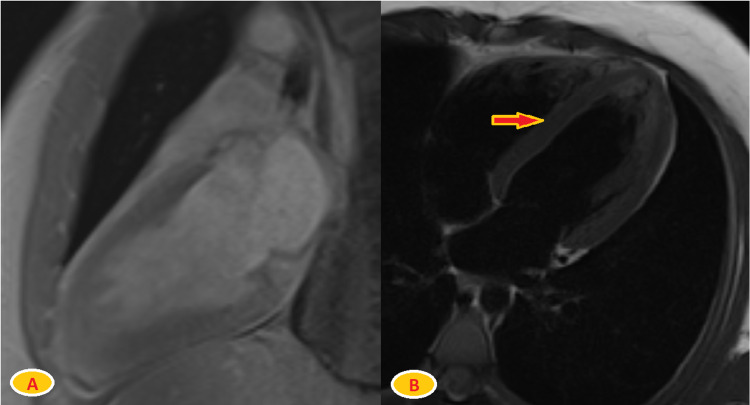
Cardiac MRI using bright blood, dark blood, T1 mapping, and delayed post-contrast signaling A) Long access post-contrast enhancement view with no Gadolinium enhancement. B) T2-weighted image with no abnormal signals in the myocardium (arrow).

The patient received sublingual nitroglycerin, which reduced his chest pain. Troponin peaked at 16 ng/mL and started trending down within 24 hours of admission. He also had elevated C-reactive protein (CRP) of 4.4 mg/dL; however, the erythrocyte sedimentation rate (ESR) of 11 mm/hour was within normal limits. To rule out other viral, bacterial, or autoimmune sources of myopericarditis, we performed the following polymerase chain reaction (PCR) infectious work-up: adenovirus, coronavirus 229E, coronavirus HKU1, coronavirus NL63, coronavirus OC43, human metapneumovirus, human rhinovirus/enterovirus, influenza A/H1 and H3, influenza A/H1-2009, influenza B, parainfluenza, respiratory syncytial virus, Bordetella pertussis/parapertussis, Chlamydophila pneumonia, Mycoplasma pneumonia, COVID-19 immunoglobulin G antibodies, HIV, parvovirus B19 PCR, immunoglobulin G, immunoglobulin M antibodies, hepatitis panel, urine drug screen, and antinuclear antibody. All tests were negative. Transthoracic echocardiography (TTE) was performed. It showed borderline left ventricular global hypokinesis and borderline reduced ejection fraction of 50-55%. The elevated troponin level and retrosternal chest pain suggested myopericarditis as the most likely diagnosis. The patient was started on lisinopril 2.5 mg once daily, colchicine 0.6 mg twice daily, and ibuprofen 800 mg every eight hours. Cardiac magnetic resonance imaging on the second after admission, using bright blood, dark blood, T1 mapping, and delayed post-contrast signaling, did not show significant myocardial edema or abnormal myocardial late Gadolinium enhancement (Figure 2).

Nonetheless, it showed a borderline reduction of ejection fraction of 53%, with minimal global hypokinesis. Prior to discharge, the patient's chest pain drastically improved. Their troponin level was <0.012 ng/mL, with CRP of 0.34 mg/dL. The patient was discharged on ibuprofen 800 mg three times daily for two weeks and colchicine 0.6 mg twice daily for three months. The case was reported to the Vaccine Adverse Event Reporting System (VAERS), which is a national system to monitor vaccines safety in the US. 

Follow-up after two weeks

**Figure 3:**
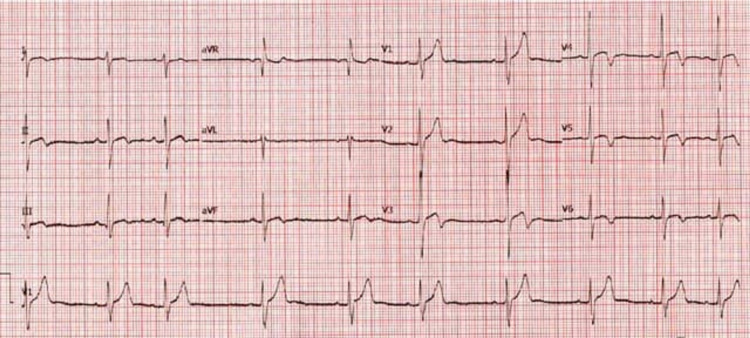
Sinus rhythm with sinus arrhythmia T-wave inversions in the anterolateral leads V3–V6

After discharge from the hospital, the patient was followed-up in the clinic. His chest pain improved significantly. The EKG showed sinus arrhythmia and T-wave abnormalities in the anterolateral leads (Figure 3).

At this follow-up, the patient had completed two weeks of ibuprofen. He was instructed to continue colchicine until the next visit in three months, to obtain ESR, CRP, and TTE tests, and to follow up with us in the cardiology clinic. Unfortunately, the patient did not follow up, and we were unsuccessful in reaching out to him.

## Discussion

Because vaccine administration is expected to continue and increase, with ongoing trials in younger populations, it is critically important to understand pharmacovigilance-related cardiovascular adverse effects. Reports of cardiac involvement, such as acute pericarditis being a sole and primary manifestation of COVID-19 infection, particularly amongst younger populations, raise the concern of a greater predisposition to adverse events in juvenile populations [5]. Cardiac injury from COVID-19 infection is reported to have an increased risk of morbidity and mortality [6]. Ammirati et al. reported a case of myocarditis that occurred three days after receiving a second dose of the BNT162b2 mRNA vaccine, with mild clinical manifestation [2]. Several other cases of myocarditis following mRNA vaccines have been reported with similar outcomes [7-9]. Acute myocarditis was not reported in both the BNT162b2 mRNA and mRNA-1273 trials. However, a few cases of ventricular arrhythmias were documented. The mRNA-1273 trial reported the deaths of two recipients, one from cardiopulmonary arrest. The BNT162b2 trial reported the deaths of four recipients, two of which were cardiac-related [4,10]. Simone et al. reported acute myocarditis after receiving COVID-19 mRNA vaccines in 5.8 cases per million [11]. A more recent analysis of a large Israeli healthcare system reported a myocarditis incidence of 2.13 cases per 100,000 persons, with a 95% confidence interval (CI) of 1.56-2.70 per 100,000. This incidence was highest among young male patients (16-29 years old), at 4.12 per 100,000 (CI 2.99-5.26 per 100,000) among those who had one or more doses of the BNT162b2 mRNA vaccine [12]. Pericarditis following vaccination has been observed with influenza and smallpox vaccinations [13,14]. In our case, the underlying mechanism of myopericarditis might be secondary to molecular mimicry or from a nonspecific inflammatory process [2]. The first theory can explain, to an extent, the high incidence of cardiac adverse effects associated with COVID-19 infection. An endomyocardial biopsy might offer better answers. However, this is not performed on a regular basis because the invasive procedure can cause serious cardiac injury and limited use across the United States [15]. The Centers for Disease Control and Prevention reported a case of severe myocarditis in which a myocardial biopsy was performed. It revealed an inflammatory infiltrate rich in T-cells and macrophages, with a mixture of B cells, plasma cells, and eosinophils [16]. In our present case, we investigated viral and autoimmune etiologies, along with less common causes of myocarditis, including HIV and hepatitis [17]. Although our patient had a negative PCR test for COVID-19 infection or reinfection, false-negative results were possible, which might have been confirmed by a later PCR test [18]. Other differential diagnoses included acute coronary syndromes, pulmonary embolism, and acute aortic syndromes. However, the patient's physical presentation and EKG results were suggestive of pericarditis and myocarditis. The onset of new symptoms after the mRNA vaccine, and the lack of other known etiologies, supported our diagnosis of COVID-19 vaccine-induced myopericarditis.

Accepted therapies for acute pericarditis include high-dose aspirin or non-steroidal anti-inflammatory drugs, colchicine, and corticosteroids (for refractory cases) [19]. Our patient had pericarditis predominantly in the spectrum of myopericarditis, as evidenced by the initial EKG and chest pain characteristics. Therefore, the main treatment consisted of ibuprofen for two weeks and colchicine for three months. There was an immediate improvement in chest pain and overall clinical indicators [20]. We anticipate a good long-term prognosis. This population of patients must be closely monitored for any unexpected outcomes.

## Conclusions

This case report highlights the importance of considering the potential side effect of cardiac injury following COVID-19 vaccination. While vaccination efforts continue to expand and include younger patients, clinicians should be aware of possible adverse cardiovascular events, such as myocarditis, pericarditis, or a combination of both (as in the present case). Recent retrospective studies have shown a primary association between mRNA COVID-19 vaccines and myocarditis/pericarditis. The benefit of vaccines substantially exceeds the infrequent mild-to-moderate side effects. There is an urgent need for further studies of large cohorts in order to establish accurate incidence rates, risk factors, and alternative treatment plans.
